# Functional analysis of genes involved in the biosynthesis of isoprene in *Bacillus subtilis*

**DOI:** 10.1007/s00253-007-0953-5

**Published:** 2007-04-26

**Authors:** Mattijs K. Julsing, Michael Rijpkema, Herman J. Woerdenbag, Wim J. Quax, Oliver Kayser

**Affiliations:** 1 Department of Pharmaceutical Biology, Graduate School for Drug Exploration (GUIDE), University of Groningen, Antonius Deusinglaan 1, 9713 AV Groningen, The Netherlands; 2 Synspec BV, De Deimten 1, 9747 AV Groningen, The Netherlands

**Keywords:** *Bacillus subtilis*, Isoprene, Methylerythritol phosphate pathway, MEP pathway, Terpenoid, Gas chromatography

## Abstract

In comparison to other bacteria *Bacillus subtilis* emits the volatile compound isoprene in high concentrations. Isoprene is the smallest representative of the natural product group of terpenoids. A search in the genome of *B. subtilis* resulted in a set of genes with yet unknown function, but putatively involved in the methylerythritol phosphate (MEP) pathway to isoprene. Further identification of these genes would give the possibility to engineer *B. subtilis* as a host cell for the production of terpenoids like the valuable plant-produced drugs artemisinin and paclitaxel. Conditional knock-out strains of putative genes were analyzed for the amount of isoprene emitted. Differences in isoprene emission were used to identify the function of the enzymes and of the corresponding selected genes in the MEP pathway. We give proof on a biochemical level that several of these selected genes from this species are involved in isoprene biosynthesis. This opens the possibilities to investigate the physiological function of isoprene emission and to increase the endogenous flux to the terpenoid precursors, isopentenyl diphosphate and dimethylallyl diphosphate, for the heterologous production of more complex terpenoids in *B. subtilis*.

## Introduction

Isoprene (2-methyl-1,3-butadiene; **I**) or actually isopentenyl diphosphate (IDP; **II**) is the general precursor of all terpenoids, that represent a very diverse class of natural products. Two evolutionary distinct routes occur in nature for the biosynthesis of IDP. The spread of the two pathways is well investigated for organisms with a sequenced genome (Lange et al. 2000; Boucher and Doolittle 2000; Rohdich et al. 2001). In eukaryotes and archaea IDP and its isomer dimethylallyl diphosphate (DMADP; **III**) are formed via the mevalonate pathway (reviewed by Kuzuyama 2002). This pathway is well studied and for many organisms the enzymes are characterized and the encoding genes identified. More recently, another pathway to IDP was discovered in some eubacteria and in plastids of higher plants, that proceeds via the intermediate methylerythritol phosphate (MEP) (Rohmer et al. 1993; Rohmer 1999).

Most Gram-negative bacteria, including *Escherichia coli*, use the MEP pathway and for *E. coli* this is now well documented. For Gram-positive bacteria the situation is less clear. Gram-positive cocci have been reported to only possess genes for the mevalonate pathway (Wilding et al. 2000). In *Streptomyces aeriouvifer* both pathways are found (Seto et al. 1996). In *Bacillus subtilis,* being regarded as the prototype for Gram-positives, only homologues of the genes for the MEP pathway are present (Wagner et al. 2000).

**Fig. 1:**
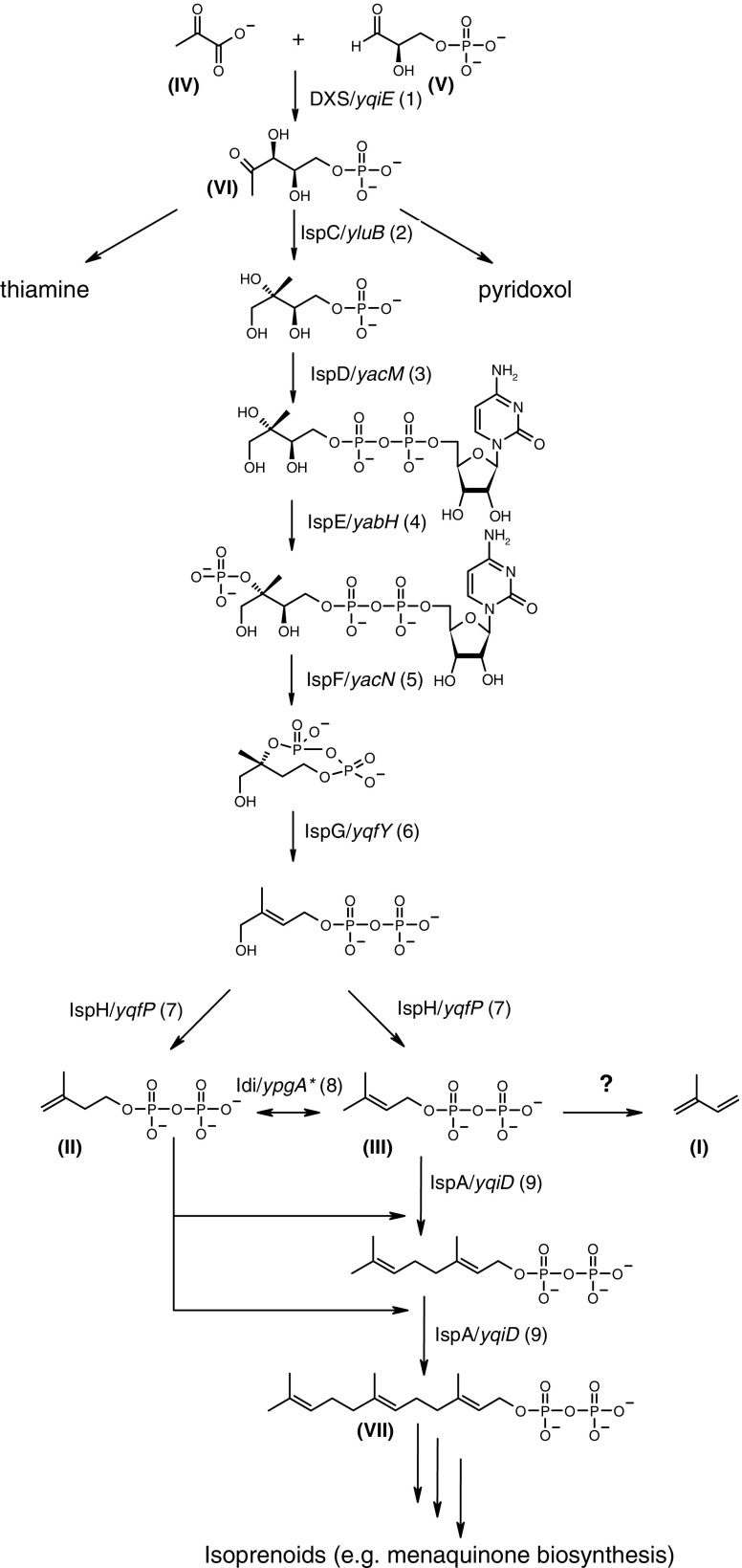
Methylerythritol phosphate pathway of isoprenoids [Dxs (1), 1 deoxy-d-xylulose 5-phosphate synthase; IspC (2), 2*C*-methyl-d-erythritol 4-phosphate synthase; IspD (3), 4-diphosphocytidyl-2*C*-methyl-d-erythritol 4-phosphate synthase; IspE (4), 4-diphosphocytidyl-2*C*-methyl-d-erythritol kinase; IspF (5), 2*C*-methyl-d-erythritol 2,4-diphosphate synthase; IspG (6), 2*C*-methyl-d-erythritol 2,4-cyclodiphosphate; IspH (7), 1-hydroxy-2-methyl-butenyl 4-diphosphate reductase; Idi (8), isopentyl diphosphate isomerase; IspA (9), farnesyl diphosphate synthase]. The putative genes are mentioned in *italics* for every biosynthetic step, including the essential gene *YpgA*, which is indicated with an *. Numbering of compounds and biosynthetic steps refers to the text

For *E. coli* the complete MEP pathway has been elucidated and the genes involved have been identified and the corresponding enzymes described (reviewed by Eisenreich et al. 2004). The pathway exists of seven subsequent enzymatic steps (Fig. 1). The first reaction of the MEP pathway is catalyzed by a transketolase (DXS) and concerns the condensation of pyruvate (**IV**) and d-glyceraldehyde-3-phosphate (**V**) to 1-deoxy-d-xylulose 5-phosphate (**VI**) (Sprenger et al. 1997; Lois et al. 1998). 1-deoxy-d-xylulose is a branching intermediate in the biosynthesis of isoprenoids, thiamine (vitamin B1) (White 1978; David et al. 1981, 1982), and pyridoxol (vitamin B6), respectively (Hill et al. 1989). The second step is catalyzed by the IspC protein, initiating a rearrangement in the carbon skeleton followed by a reduction in the keto function of (**VI**), and finally delivering 2*C*-methyl-d-erythritol 4-phosphate (Takahashi et al. 1998). Five other subsequently acting enzymes are needed for the synthesis of IDP, including a phosphocytidyl transferase (IspD), a kinase (IspE), a cyclophosphate synthase (IspF), and two reductases (IspG, IspH) (Fig. 1).

Bacteria use the precursors IDP and DMADP for the synthesis of several compounds including the side chains of ubiquinone or menaquinone. Next to that, several bacterial species use these precursors to synthesize isoprene, that is emitted to their environment (Kuzma et al. 1995). *B. subtilis* emits isoprene in high levels compared to other bacterial species as has been described for *B. subtilis* 6051, *B. subtilis* 23059, and *B. subtilis* 23856 (Kuzma et al. 1995). Using a bioreactor system, the emission of isoprene by *B. subtilis* 6051 was found to occur in three phases during the growth curve (Wagner et al. 1999). The phases correspond with respectively, glucose metabolism and secretion of acetoin, catabolism of acetoin, and the early stage of sporulation. The general laboratory strain *B. subtilis* 168 showed another pattern of isoprene emission, lacking phases 2 and 3 (Fall and Copley 2000). The function of the emitted isoprene has been postulated as being a signal molecule in the natural environment of the microorganism. Another possible explanation for the emitted isoprene is the efflux as an overflow metabolite in the bacterial pathway to isoprenoid structures (Fall and Copley 2000). The uptake of isoprene by microorganisms present in soil samples has been described as a sink of atmospheric isoprene (Cleveland and Yavitt 1998). However, there is no full evidence supporting one of the hypotheses mentioned. Isoprene originates from DMADP. The conversion of DMADP in isoprene is known to be an enzymatic process in the poplar tree from which a gene has been isolated and characterized (Miller et al. 2001; Schnitzler et al. 2005). Attempts to prove the enzymatic conversion to isoprene in *B. subtilis* suggested the involvement of an enzymatic step. Enzymatic activity has been partially purified, but the enzyme turned out to be very labile (Sivy et al. 2002). Until now the encoding gene could not be identified. A search for a homologue protein of the isoprene synthase from poplar tree in the genome of the *B. subtilis* did not yield a candidate. Based on homology with known genes, mostly from *E. coli*, candidate genes for the other enzymatic steps in the MEP pathway in *B. subtilis* have been suggested. Identification of the genes involved in this pathway should shed some light on the function of isoprene synthesis in *Bacillus* physiology. Due to the essentiality of the products downstream of the isoprenoid biosynthesis, like menaquinone, it is not remarkable that the candidate genes, except for the isomerase, have been shown to be essential genes for the survival of the bacterial cell (Kobayashi et al. 2003). In the construction of the BFAN collection these knock-out strains were not viable and therefore conditional mutants have been made using a pMUTIN3-vector. The expression in these strains can be regulated by the isopropyl-beta-d-thiogalactopyranoside (IPTG) inducible Pspac promoter (Yansura and Henner 1984; Vagner et al. 1998). Using this mutant system, expression levels can be regulated to a minimum for survival by decreasing the amounts of IPTG. In that way, the function of the genes can be investigated. Identification of the involvement of certain genes in the isoprenoid biosynthetic pathway, may create possibilities to redirect the metabolic flux toward IDP. The possibilities of pathway engineering have shown their strength already in other microorganisms, like *E. coli* and *Saccharomyces cerevisiae*. The metabolic flux was dependent mainly on the deoxyxylulose 5-phosphate synthase (DXS), the isopentenyl diphosphate isomerase (Idi), and the prenyltransferase delivering farnesyl diphosphate (IspA) (Harker and Bramley 1999; Kim and Keasling 2001; Kajiwara et al. 1997; Martin et al. 2003). By up-regulation of the MEP pathway, *B. subtilis* can be developed as an interesting host organism for the production of complex terpenoid compounds, such as the valuable drugs artemisinin and paclitaxel. In the present study we describe the functional analysis of genes in *B. subtilis* putatively encoding enzymes involved in the biosynthesis of the terpenoid precursor isopentenyl diphosphate. We used quantitative isoprene emission of different well controllable knock-out *Bacillus* strains in their environment to determine the function of the tested genes.

## Materials and methods

### Bacterial strains and media

Bacterial strains used are listed in Table 1. *B. subtilis* strains 6051, 23059 and 23856 were obtained from the American Type Culture Collection (ATCC) (Rockville, USA). *B. subtilis* mutant strains were obtained through a chromosomal integration of pMUTIN3 derived plasmids (Vagner et al. 1998). Experiments were performed using Luria-Bertani (LB)-medium containing 1% bacto-tryptone, 0.5% yeast extract, and 0.5% NaCl (all purchased from Duchefa, Zwijndrecht, the Netherlands). If required, medium was supplemented with erythromycin (2 μg/ml; Duchefa, the Netherlands) or the specified concentration of isopropyl-β-d-thiogalactopyranoside (IPTG; Duchefa, the Netherlands). For growing on plates the medium was solidified with 1.5% agar.

**Table 1:** Putative genes for the MEP pathway in the genome of *B. subtilis* and the percentage of identities with known proteins from *E. coli*, the essentiality of each gene, the corresponding BFAN mutant strain (explained in detail in the text, with numbering of the respective enzymatic steps)

Enzyme	*E. coli*	*B. subtilis*	Essential gene	Mutant strain
Dxs (1)	*dxs*	*dxs/yqiE* (43%)	+	168I*yqiE*
IspC/Dxr (2)	*dxr*	*yluB* (43%)	+	168I*yluB*
IspD (3)	*ygbP*	*yacM* (36%)	+	168I*yacM*
IspE (4)	*ychB*	*yabH* (27%)	+	168I*yabH*
IspF (5)	*ygbB*	*yacN* (56%)	+	168I*yacN*
IspG (6)	*gcpE*	*yqfY* (46%)	+	168I*yqfY*
IspH (7)	*lytB*	*yqfP* (35%)	+	168I*yqfP*
Idi I	*idi*	–		–
Idi II (8)	–	*ypgA* (39%)^a^	–	168*ypgA*
IpsA/FPPS (9)	*ispA*	*yqiD* (43%)	+	168I*yqiD*

^a^Homologue of *Streptomyces* protein (Kaneda et al. 2001)

### Detection of bacterial isoprene emission

A single colony of the different *B. subtilis* strains was transferred from a plate to 10 ml Luria-Bertani (LB) medium (if required supplemented with 2 μg/ml erythromycin and 100 μM IPTG) and grown over night (37°C; 300 rpm). Before the inoculation (1:100) of fresh LB medium containing different specified concentrations of IPTG, cells were gently washed three times with fresh LB-medium without IPTG by resuspending and centrifugating.

Isoprene accumulation was measured on-line by sampling every 15 min, during a period of nine hours, 15 ml of the air above 50 ml bacterial culture, growing (37°C; 300 rpm) in a 500 ml Erlenmeyer air tight flask (CBN, the Netherlands), and transferring into a gas-chromatography system suitable for the sensitive detection of isoprene (Syntech Spectras GC955 series 601, Synspec BV, the Netherlands) (Loreto and Delfine 2000). The air was pumped through a Tenax GA trap, desorbed at 180°C and transferred to an AT 5 column under a flow of 2.5 ml/min nitrogen (3.7 bars; quality 5). The temperature program used was 3 min at 50°C followed by an increase in temperature to 70°C at 5 min; kept at this temperature until 12 min and than lowered to 50°C again. The isoprene present was detected by photo ionization at 10.6 eV. The gas chromatograph was calibrated using the dynamic gas dilution principle with several concentrations of gaseous isoprene using liquid isoprene (Sigma, USA) diluted in methanol and evaporated with a gas dilutor (MK5, MCZ Umwelttechnik, Germany). During the isoprene detection the growth of the bacterial culture was determined by measuring the optical density at 600 nm (OD_600_ _nm_) every hour.

## Results

### Isoprene emission in wild-type *Bacillus* strains

The emission of isoprene from *B. subtilis* wild type strains 168, 6051, 23059, and 23856 was investigated. For all four strains isoprene accumulated in the logarithmic phase of growth, leading to a high increase in isoprene in the flask. The absolute maximum of wild type strains varied between 400 and 700 μg/m^3^. Starting from the late logarithmic phase to the beginning of the stationary phase the amount of isoprene in the flask decreased slowly.

**Fig. 2:**
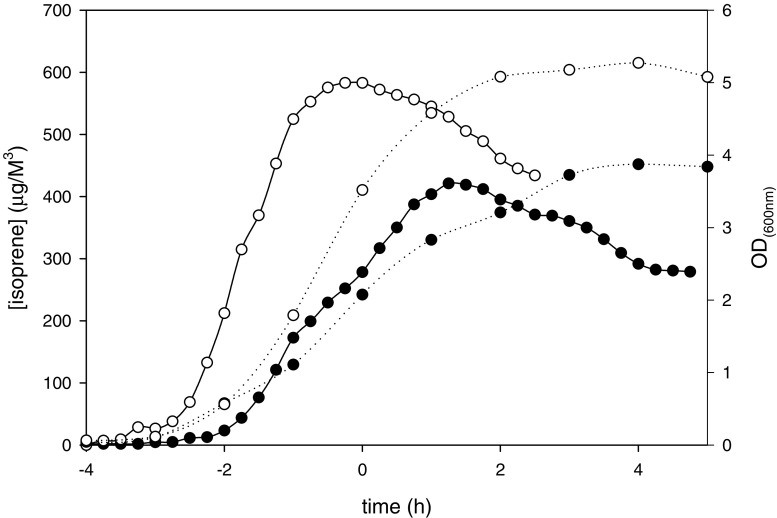
Isoprene emitted (solid lines) by wild type strains *B. subtilis* 168 (•) and *B. subtilis* 6051 (○) during growth (dashed lines). Zero time (t = 0) indicates the transition point between the exponential and the post-exponential growth phases

Figure 2 shows the accumulation of isoprene during the growth of the wild type strains 168 and 6051. *B. subtilis* 6051 reached higher levels of isoprene, but this is probably not caused by a higher production rate rather than the amount of cells present, as reflected by the optical density of these strains. Corrected for the amount of cells, determined as the optical density at 600 nm, *B. subtilis* 168 accumulated at maximum 148 μg/ m^3^ / OD_600_ _nm_ compared to 166 μg/ m^3^ / OD_600_ _nm_ for *B. subtilis* 6051. The other two wild type strains tested, 23059 and 23856, showed a similar accumulation of isoprene as the strains described before. Corrected for the amount of cells, 23059 produced 192 μg/ m^3^ / OD_600_ _nm_ and 23856 produced 112 μg/ m^3^ / OD_600_ _nm_ isoprene at maximum accumulation (Table 2).

**Table 2:** Maximum concentration of isoprene accumulated after emission by the different wild type *Bacillus subtilis* strains corrected for the amount of cells (OD_600_ _nm_)

*Bacillus subtilis* strain	Maximal isoprene accumulation (μg/m3/OD_600_ _nm_)
168	149
6051	166
23059	192
23856	111

### Mutant strains

To find the optimal conditions in which expression of the genes can be limited without killing the cells, each conditional mutant strain was subjected to IPTG depletion by growing the strains on agar plates containing different concentrations of IPTG varying from 0 to 1 mM. Decreasing the concentration of IPTG caused growth inhibition after overnight incubation resulting in smaller and finally no colonies (on plates without IPTG) at 37°C. Table 3 shows the minimal concentration of IPTG at which still some growth was observed for the different conditional mutant strains. This concentration of IPTG was used in our experiments and defined as the condition causing limited induction. Most mutant strains indeed showed growth inhibition at lower concentration of IPTG. The mutant strain168I*yqfY*, the mutant for the putative gene encoding a homologue of the IspG protein (step 6) did not show any dependency on IPTG on plates (varying IPTG concentrations from 0 to 1 mM). Plates without IPTG incubated overnight resulted in the growth of normal colonies and the liquid cultures obtained growth curves comparable with wild type *B. subtilis* 168, independent of the concentration of IPTG. For some of the other mutant strains there was some growth of the bacterial cultures without IPTG as well, although severely impaired.

**Table 3:** Maximum amount of isoprene accumulated after emission by the different mutant *Bacillus* strains, corrected for the amount of cells (OD_600_ _nm_)

*Bacillus* strain	Limited expression (μM IPTG)	Maximal isoprene accumulation (μg/m3/OD_600_ _nm_)			Relative decrease
		Limited induction	Full induction	Full repression	
168I*yqiE* (1)	10	8	119	27	15.2
168I*yluB* (2)	1	31	167	13	5.4
168I*yacM* (3)	50	4	113	4	27.9
168I*yabH* (4)	25	29	160	7	5.6
168I*yacN* (5)	10	14	119	20	8.8
168I*yqfY* (6)	–	n.d.	194	147	–
168I*yqfP* (7)	1	13	130	17	9.8
168*ypgA* (8)	–	–	–	103	–
168I*yqiD* (9)	10	122	166	139	1.4

The relative decrease is calculated as the maximal amount of isoprene at full induction devided by the maximal amount of isoprene at limited induction (dashes indicate that measurement did not apply here; explained in detail in the text, with numbering of the respective enzymatic steps).

*n.d.*, not determined

For all conditional knock out strains isoprene emission was measured at the concentration of IPTG with limited expression in comparison to 1 mM (full induction) and no IPTG (full repression).

**Fig. 3:**
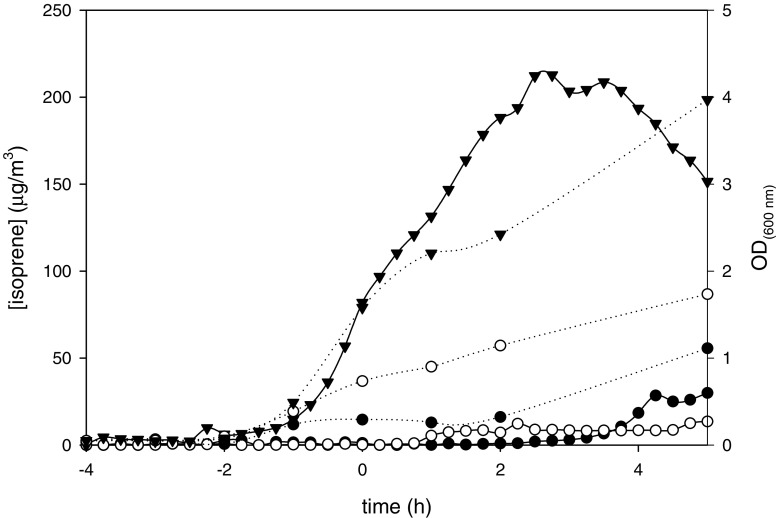
Isoprene emitted (solid lines) by the *B. subtilis* mutant 168IyqiE during growth (dashed lines) in the presence of 1 mM IPTG (▾), 10 μM IPTG (○), and no IPTG (•). Zero time (t = 0) indicates the transition point between the exponential and the post-exponential growth phases

Figure 3 represents the data for strain 168I*yqiE*, the conditional mutant strain for the first step in the biosynthetic pathway. Full induction of the *dxs* gene resulted in a normal growth curve of the bacterial culture and in an isoprene accumulation profile comparable to the profile of the wild type stain *B. subtilis* 168. Depletion of IPTG inhibited cell growth. The maximal concentration of isoprene accumulated in the flask and corrected for the amount of cells was 15 fold lower for the culture supplemented with 10 μM IPTG (8 μg/ m^3^ / OD_600_ _nm_) than for the culture with 1 mM IPTG (119 μg/ m^3^ / OD_600_ _nm_).

Table 3 shows the maximum concentration of isoprene accumulated in the flask calculated on the amount of cells present at that time point for all tested mutant strains. Mutant strains for putative genes *yluB*, *yacM*, *yabH*, *yacN*, and *yqfP* encoding enzymes for respectively, steps 2, 3, 4, 5, and 7 showed significant differences in isoprene emission, varying from a 5–28 fold decrease at limited expression levels in comparison to full induction.

The knock-out strain of *ypgA*, putatively encoding an isopentenyl diphosphate isomerase (step 8), is the only knock-out of a nonessential gene. The mutant strain showed a normal growth curve and did not show a significant difference in the emission of isoprene in comparison to the wild type strain *B. subtilis* 168.

We also tested the function of *yqiD*, encoding a homologue for farnesyl diphosphate synthase (FDP; **7**), synthase IspA (step 9) of *E. coli*. Depletion of IPTG to 10 μM did not result in a significant change in the isoprene emission by the mutant strain, while it did show to be dependent on IPTG for growth.

## Discussion

To identify genes involved in the biosynthesis of the highly volatile compound isoprene, an efficient online detection system for this terpenoid (precursor) in the air above bacterial cultures was developed. Since the *B. subtilis* strains 6051, 23059, and 23856 were known to emit isoprene (Kuzma et al. 1995), we used these strains to set up the detection system for *B. subtilis* 168 for which the genome has been sequenced (Kunst et al. 1997) and functional knock outs have been made. All four strains emitted isoprene in our experiment. The differences in the amounts of isoprene emitted corresponded with the levels as described before, where *B. subtilis* 6051 and *B. subtilis* 23059 emitted higher amounts than *B. subtilis* 23856 (Kuzma et al. 1995). Since *B. subtilis* 168 showed emission of isoprene, the use of the experimental set up to investigate the isoprene synthesis by the mutant strains was justified. From the profile of isoprene emission with a maximum at the transition of the growth curve from the logarithmic phase to the stationary phase, it was concluded that from this time point on maximum isoprene concentrations had been reached and that amounts sampled from the culture every 15 min exceeded the production of new isoprene. We could not detect isoprene production in three phases using standard incubation in a shaking flask as has been described for *B. subtilis* 6051 grown in a bioreactor.

The conditional knock out strain of the *yqiE* encoding DXS, the transketolase responsible for the first step in the biosynthesis of isoprenoids, appeared to be highly dependent on IPTG. The dramatic decrease in isoprene production at low concentration of IPTG supported the role of this gene in the biosynthetic pathway of isoprene. *yqiE* has been identified as *dxs* encoding 1-d-deoxyxylulose-5-phosphate in *B. subtilis* before (Harker and Bramley 1999; Hecht et al. 2001). However, the confirming results for this mutant strain proved that the experimental set up of our work was valid to investigate the function of the other putative genes. Apparently, the isoprene emission is severely decreased when genes in the pathway are expressed at lower level than in the wild type strain.

Growth at the end of the incubation after several hours, as shown for many of the liquid cultures under IPTG depletion or even without suppletion of IPTG, may be explained by the occurrence of reversions in the mutant strain (Zanen et al. 2006). Another explanation could be the presence of a not fully repressed promoter in the complementation strain. It has been reported that in the used vector pMUTIN3 the Pspac promoter can give some low expression even in the absence of IPTG (Vagner et al. 1998; Petit et al. 1998). For genes showing low levels of expression in the wild type strain it is easy to get a pseudo wild type level of expression in the mutant strain. This may be in particular the case for highly efficient enzymes in the biosynthetic pathway.

For the putative genes *yluB*, *yacM*, *yabH*, *yacN*, and *yqfP*, encoding homologues of the enzymatic steps 2, 3, 4, 5 and 7, the isoprene accumulation of the conditional knock outs by depletion of IPTG clearly proved the involvement of these genes in the biosynthetic pathway isoprene. *B. subtilis* 168I*yqfY* (step 6) did not depend on IPTG and therefore nothing can be concluded from the data obtained with this mutant strain other than that this enzyme may be highly efficient leading to complementation already obtained at the low expression levels caused by leaking activity of the Pspac promoter (Zanen et al. 2006).

The nonessential gene *ypgA* encodes a homologue protein for an isopentenyl diphosphate isomerase (Idi) (step 8). The *B. subtilis* gene *ypgA* encodes for a so-called isopentenyl diphosphate isomerase type II protein (Takagi et al. 2004), while the genome of *E. coli* harbours a gene for a different Idi type I enzyme (Hahn et al. 1999). Interestingly, all archaea contain the Idi type II and all eukaryotes contain Idi type I. In genomes of eubacteria both are found, but there are also genomes without any of the described genes (Boucher and Doolittle 2000). In contrast to all other genes investigated in this study, the gene *ypgA* encoding for the IdiII was shown to be a nonessential gene in *B. subtilis* (Takagi et al. 2004). We also observed that the knock-out mutant of the bacterial strain is viable and produces isoprene. The *idiI* gene of *E. coli* has also been shown to be nonessential. Deletion mutants of this gene were viable on minimal medium (Hahn et al. 1999). Our findings, that the isoprene emission is not influenced by the knock out of the *ypgA* gene, can be explained by the enzymatic mechanism of the IspH protein, step 7, before the isomerase. IDP and DMADP can be synthesized independently by the catalytic action of IspH. A hypothetical mechanism for this reaction has been described (Eisenreich et al. 2004). This supports the nonessential character of the gene. The presence of isoprene proves that the isomer DMADP is still synthesized in the mutant and that the cells do not depend on the isomerase for the production of DMADP from IDP only. The isomerase functions in the balance of IDP and DMADP as a salvage protein (Eisenreich et al. 2004).

The first enzymatic biosynthetic step downstream the formation of the precursors IDP and DMADP is catalyzed by IspA, farnesyl diphosphate synthase (step 9). Therefore the putative gene is not involved in the biosynthesis of isoprene itself. The observation that isoprene is still emitted while the expression of the gene was depleted is easily explained by its function in the pathway downstream of IDP biosynthesis. Although some accumulation of the precursors IDP and DMADP could be expected by blocking *yqiD*, no significant differences in the flux toward the isoprene emission were detected. Apparently the efflux of isoprene is not influenced by the changes in FDP synthesis downstream in the isoprenoid pathway. This can be regarded as a contradiction to the hypothesis that isoprene emission functions as an efflux of an overflow metabolite.

In this study the involvement of several *B. subtilis* genes in the MEP pathway to isoprene was established by studying isoprene emission of mutants. Five genes, essential for viability, *yluB*, *yacM*, *yabH*, *yacN*, and *yqfP,* were shown to be essential for the isoprene production as well. Where these genes were at first instance depicted based on homology of the encoding proteins, the results of this study proved the functional involvement of the genes in the biosynthesis of isoprenoids on a biochemical level. A knockout of the sixth candidate gene, *yqfY*, did not yield a reduction in isoprene emission, nor a growth retardation, which might be explained by a not fully repressed Pspac promoter present in vector pMUTIN3. Knock-outs of the two other genes investigated in this study, showing no growth retardation as well as no reduction in isoprene emission, behaved in accordance with expectation.

Although it remains unknown why isoprene is emitted by bacteria, the knowledge about the pathway to isoprene can be used for further investigations towards a better understanding of the metabolic flux to IDP and its physiological function. Next to that the IDP pool can be optimized by metabolic pathway engineering, creating a *B. subtilis* strain as an efficient Gram-positive host for the heterologous production of terpenoids, like the valuable plant-derived pharmaceuticals artemisinin and paclitaxel. The potential of using heterologous production organisms for the supply of terpenoids is already under investigation for *E. coli* and for several yeast strains (Martin et al. 2003; Lindahl et al. 2006; Ro et al. 2006; Dejong et al. 2006). For the purpose of metabolic pathway engineering it is interesting to search for a gene encoding isoprene synthase in *B. subtilis* as well. Evidence for an enzymatic bioconversion and the identification of this specific enzyme could give the possibility to block the efflux of isoprene thereby increasing the amount of isoprenoid available for the synthesis of terpenoids.
